# Relation Between HbA1c and Lipid Profile Among Prediabetics, Diabetics, and Non-diabetics: A Hospital-Based Cross-Sectional Analysis

**DOI:** 10.7759/cureus.32909

**Published:** 2022-12-24

**Authors:** Sushil Kumar, Bandana Kumari, Amit Kaushik, Ayan Banerjee, Mala Mahto, Akash Bansal

**Affiliations:** 1 Biochemistry, All India Institute of Medical Sciences, Patna, IND; 2 Community Medicine, Dr. Ram Manohar Lohia Institute of Medical Sciences, Lucknow, IND

**Keywords:** poor glycemic control, triglyceride, vldl, prediabetics, lipid profile, dyslipidemia, hba1c, diabetes

## Abstract

Introduction: An unusually high blood glucose level is a hallmark of diabetes mellitus, with an imbalance between insulin levels and insulin sensitivity leading to an insulin functional deficit. Since it serves as both a risk indicator and a gauge of long-term glycemic control, the HbA1c concentration is a crucial component of standard diabetes treatment. The use of the HbA1c concentration in the diagnosis of diabetes is expanding as the test's accuracy increases. Dyslipidemic profiles can appear before type 2 diabetes manifests itself and are independent risk factors for the disease. Additionally, dyslipidemia, especially in diabetics, might affect pancreatic beta-cell survival and activity. This study was undertaken with the aim to find out any correlation between HbA1c and lipid profile among diabetics, prediabetics, and non-diabetics.

Methods: A total of 1,000 individuals with age 18-60 years were included in the study (non-diabetics = 186, prediabetics = 238, diabetics = 576). HbA1c was estimated by capillary electrophoresis and a lipid profile was done using a fully automatic chemistry analyzer.

Result: Diabetes was found to be significantly associated with dyslipidemia. In diabetics, a statistically significant increase in the level of triglyceride and very low-density lipoprotein (VLDL) was seen as compared to prediabetics. Diabetic women were found to be significantly more dyslipidemic as compared to diabetic males. The mean HbA1c among diabetics was found to be 8.3.

Conclusion: In hyperglycemia-induced dyslipidemia, raised triglyceride and VLDL were the most common findings, and combined lipid abnormalities were more commonly seen as compared to a single abnormality in the lipid profile. Patients with poor glycemic control more commonly develop dyslipidemia, which may be a reason for an increased incidence of cardiovascular catastrophes in such patients.

## Introduction

Diabetes mellitus (DM) is a metabolic disorder characterized by abnormally high blood glucose levels. Type 1, type 2, maturity-onset diabetes of the young (MODY), gestational diabetes, neonatal diabetes, and secondary causes related to endocrinopathies, steroid usage, and other factors are all types of diabetes [1]. Around one in every 11 persons in the globe now has diabetes, with 90% of those suffering from type 2 diabetes (T2DM) [2]. T2DM has a more gradual start, with an imbalance between insulin levels and insulin sensitivity leading to an insulin functional deficit. Insulin resistance can be caused by a variety of factors, although it is most typically caused by fat and aging [1]. Type 2 diabetes is becoming more common as people age and obesity rates rise, and it is now the ninth leading cause of death worldwide [3]. Glycated hemoglobin (HbA1c), a type of hemoglobin that indicates an individual's three-month average plasma glucose concentration [4], has been recommended by the American Diabetes Association as an alternative to glucose tolerance testing for diagnostic surveillance of diabetes and prediabetes since the early 21st century. The HbA1c concentration is an essential aspect of normal diabetes therapy since it is a risk predictor and a measure of long-term glycemic control. The HbA1c concentration is becoming more widely used in the diagnosis of diabetes as the test's quality improves [5]. Dyslipidemic profiles are independent risk factors for type 2 diabetes, according to epidemiological research, and can emerge before the illness develops [6,7]. Furthermore, dyslipidemia, particularly in diabetic individuals, can result in impaired pancreatic beta-cell activity and survival [6,8,9]. The link between HbA1c, dyslipidemias, and other risk factors for type 2 diabetes is not well known. According to a study by Arshad et al., HbA1c can be used as a predictor of dyslipidemia, in addition to a valid glycemic index, and thus the early diagnosis of dyslipidemia can be employed as a preventive approach for the development of CVD in patients with T2DM. This study was designed to study and understand if there lies any correlation between HbA1c and lipid profile among diabetics, prediabetics, and non-diabetics.

## Materials and methods

The present study was accomplished at a tertiary care center in eastern India, and the protocol was approved by the Institute Research Committee and Institute Ethics Committee (IRC/IEC) with an approval letter number AIIMS/Pat/IRC/2022/889 and AIIMS/Pat/IEC/2022/889, respectively. It was conducted between April 2018 and December 2019. During this study period, all the 1000 people who gave samples for blood sugar estimation and consented to participate in the study were included consecutively, out of which 186 were non-diabetic, 238 were pre-diabetics and 576 were new cases of diabetes. Cases were diagnosed as DM type - 2 (DM-2) as per the American Diabetes Association criteria 2007. Patients with DM type 1 (DM-1), pregnancy, thyroid disorders, renal problems, and other endocrinopathies, patients on lipid-lowering agents, and with a serum triglyceride (TG) > 400mg/dL were excluded from the study.

Diagnosed diabetic patients in the age group of 18-60 years comprising the diabetic group were subjected to blood sampling for estimation of fasting and postprandial blood glucose, glycated hemoglobin, and lipid profile. Other relevant data pertaining to demographic profile, clinical history, laboratory parameters, and drug history were recorded. The results of blood tests were obtained from the electronic medical records of the Hospital Information System (HIS).

From all the subjects, after an overnight fast, venous blood samples (except postprandial glucose) were collected in the central sample collection center AIIMS Patna in an appropriate amount and an appropriate vacutainer (plasma glucose in the gray cap, for glycated hemoglobin in a purple cap, and for lipid profile in red cap). The postprandial blood sample was collected in a gray tube after two hours of breakfast.

All the biochemical analyses were done in the Biochemistry laboratory of the central laboratory of AIIMS Patna. Samples for glycated hemoglobin were processed by capillary electrophoresis on Minicap flex piercing (Sebia, France). Lipid profile samples were processed by the principle of photometry on a fully automated clinical chemistry analyzer AU 680, Beckman Coulter, Japan, using kits supplied by Randox laboratories limited, UK. Serum total cholesterol was done by Enzymatic endpoint method Rx Suzuka; Triglycerides were processed by GPO-PAP method Rx Suzuka and HDL-cholesterol was processed by Direct Clearance Method Rx Suzuka. LDL was calculated by the Frieldwald equation formula and very low-density lipoprotein (VLDL using the formula TG/5.

**Table 1:** Reference range of lipid profile as per The National Cholesterol Education Program (NCEP) Adult Treatment Panel III (ATP) LDL - Low Density Lipoprotein; HDL - High Density Lipoprotein; Triglycerides/5

LDL Cholesterol									
Optimum	Near or above optimum			Borderline		High			Very high
<100 mg/dl	100-129 mg/dL			130-159 mg/dL		160-189 mg/dL			>190 mg/dl
HDL Cholesterol									
Low					High				
<40 mg/dL					>60 mg/dl				
Triglyceride									
Desirable			Borderline				High		
<150 mg/dL			150-199 mg/dL				>200 mg/dL		
Total Cholesterol									
Desirable		Borderline						High	
<200 mg/dL		200-239 mg/dL						>240 mg/dL	

Criteria used for the selection of the sample

Newly diagnosed DM-2 patients attending the sample collection center with their specific identity number, demographic data, clinical history, drug history, treatment history, and other data recorded from the patient’s clinical booklet (n=1,125). The results of laboratory tests were retrieved from electronic medical records via HIS. On the basis of exclusion criteria, only 814 patients' data were enrolled in the study.

Non-diabetic patients attending the sample collection for blood investigation for plasma glucose (fasting, postprandial random), glycated hemoglobin, and lipid profile were selected. Specific identity numbers, demographic data, History, drug history, treatment history, and other data are recorded from the patient’s clinical booklet. On the basis of exclusion criteria, data has been selected for the study as a control (n=186).

All the requisite information was collected by using pretested standard proforma by trained persons. At regular intervals, random proformas were cross-checked by the investigator for correctness and completeness. Information collected was entered into an MS excel sheet and coded. Double data entry was performed to verify the correctness of the data being entered for the information of the 10% proforma. SPSS version 23.0 and R software were used for data analysis and to prepare graphs. Proportions were used for qualitative data while mean and standard deviation were calculated for quantitative data. To study the association between categorical variables chi-square test was used while for the numerical variables t-test, ANOVA, and Bonferroni post hoc analysis were used.

## Results

The mean age of the diabetic subjects in the current study was 52.8 years. As revealed in Table 1, the mean HbA1c among diabetics was 8.3. The lipid profile of the study subjects is also shown in Table 2.

**Table 2:** Mean values of age, HbA1c, and lipid profile among prediabetics/diabetics (n = 814, HbA1c ≥5.7). Hba1c, glycated hemoglobin; TG, triglyceride; HDL, high density lipoprotein; VLDL, very low-density lipoprotein; LDL, low density lipoprotein Normal reference range for Glycated Hb (HbA1c) - 4% to 5.6%. Prediabetic HbA1c- 5.7% to 6.4%. Diabetic HbA1c - 6.5% & above

S. No.	Variable	Mean±SD
1	Age (Years)	52.8±12.1
2	HbA1c (%)	8.3±2.6 %
3	TG	151.9±70.1 mg/dl
4	Cholesterol	180.0±45.0 mg/dl
5	HDL	43.0±12.0 mg/dl
6	VLDL	30.3±14.0 mg/dl
7	LDL	106.3±35.5 mg/dl

As revealed in Table 3, it is evident that the percentage of study participants with dyslipidemia was higher among those who had diabetes (HbA1c ≥6.5) as compared to their counterparts in the prediabetic stage (HbA1c 5.7-6.4). The association of dyslipidemia (TG>150, CHOL >200, VLDL>30 and LDL>130) was found to be statistically significantly associated with diabetes. No statistically significant association was revealed between diabetes and levels of HDL among study subjects.

**Table 3:** Lipid profile with desirable vs borderline high lipid profiles among prediabetics and diabetics TG, triglyceride; HDL, high density lipoprotein; VLDL, very low density lipoprotein; LDL, low density lipoprotein; CHOL, total cholesterol

S. No	Parameter	HbA1c 5.7-6.4 (n=238)	HbA1c ≥6.5 (n=576)	Test of significance (P-value)
1	TG (mg/dL) Up to 150	156 (65.5)	321 (55.7)	P=0.01
	>150	82 (34.5)	255 (44.3)	
2	CHOL (mg/dL) Up to 200	180 (75.6)	393 (68.2)	P=0.03
	>200	58 (24.4)	183 (31.8)	
3	HDL (mg/dL) >40	135 (56.7)	329 (57.1)	P=0.9
	Up to 40	103 (43.3)	247 (42.9)	
4	VLDL (mg/dL) Up to 30	156 (65.5)	321 (55.7)	P=0.01
	>30	82 (34.5)	255 (44.3)	
5	LDL (mg/dL) Up to 130	193 (81.1)	430 (74.7)	P=0.04
	>130	45 (18.9)	146 (25.3)	

**Figure 1:**
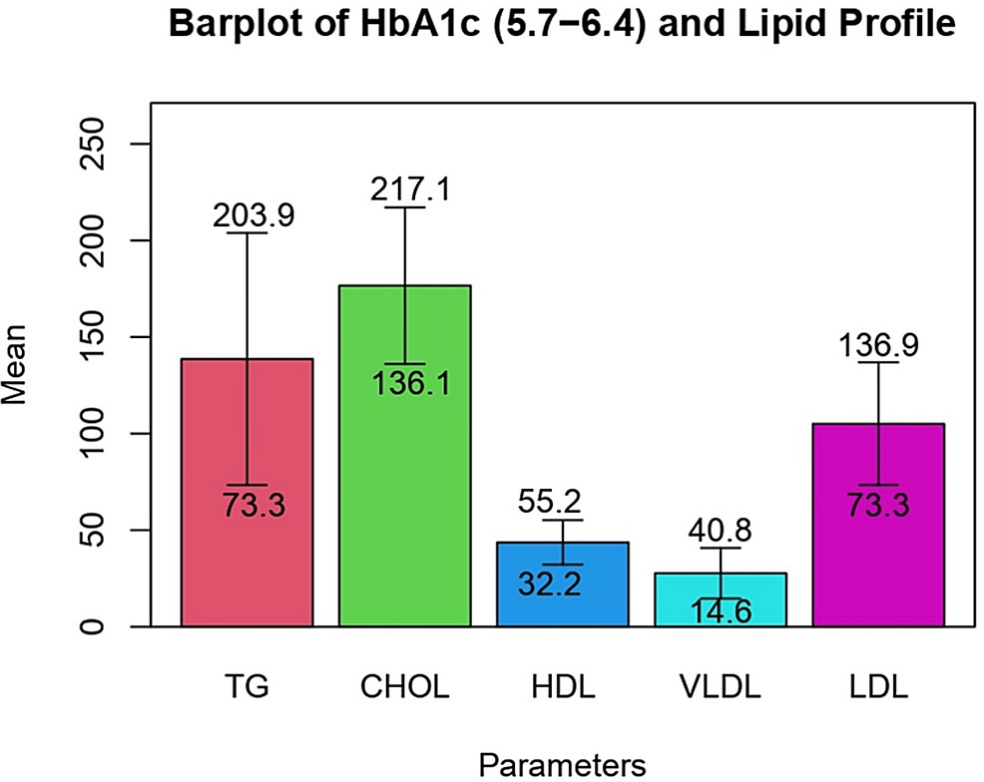
Bar plot representation of mean values of lipid profile in prediabetic subjects TG, triglyceride; HDL, high density lipoprotein; VLDL, very low-density lipoprotein; LDL, low density lipoprotein; CHOL, total cholesterol

**Figure 2:**
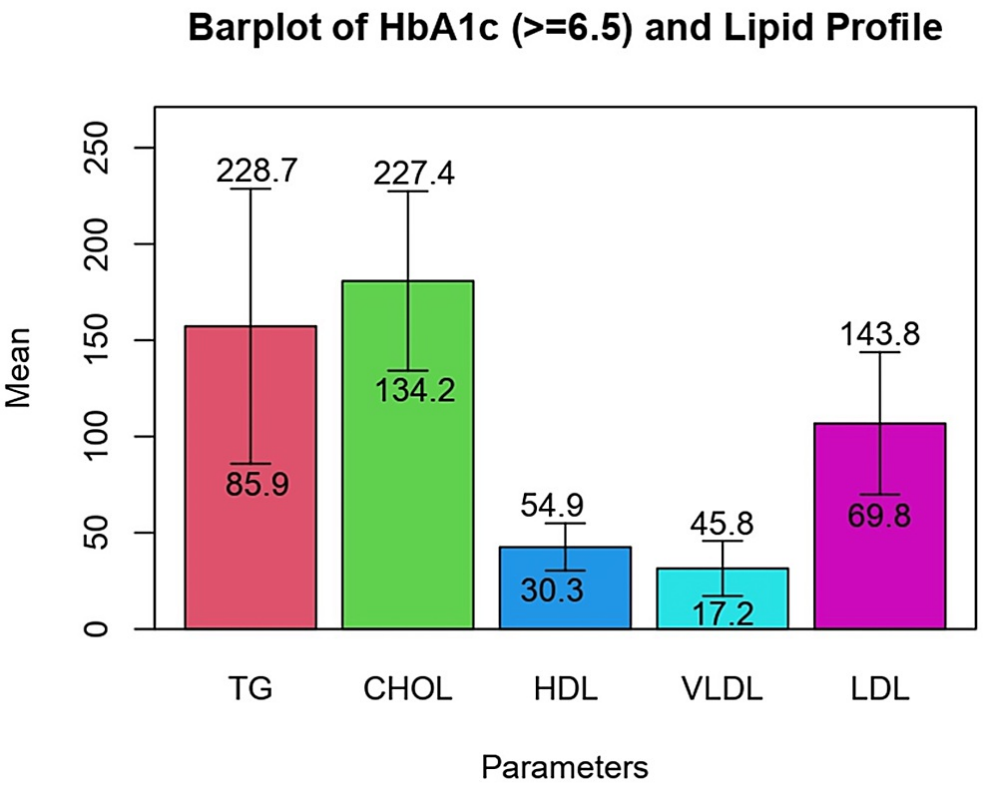
Bar plot representation of mean values of lipid profile in diabetic subjects TG, triglyceride; HDL, high density lipoprotein; VLDL, very low-density lipoprotein; LDL, low density lipoprotein; CHOL, total cholesterol

The levels of triglycerides, total cholesterol, VLDL, and LDL were found to be higher among those who had diabetes as compared to those in the prediabetic phase. The association between mean levels of TG and VLDL with glycemic control among study participants was revealed to be statistically significant. Although an inverse relationship was found between mean HDL value and glycemic control, this association was not statistically significant. (Table 4) Bar plot representation gives a better graphical picture of the given findings in prediabetic and diabetic subjects (Figures 1, 2).

**Table 4:** Mean values of lipid profile as observed in prediabetics and diabetics TG, triglyceride; HDL, high density lipoprotein; VLDL, very low-density lipoprotein; LDL, low density lipoprotein; CHOL, total cholesterol

S. No	Parameter	HbA1c 5.7-6.4%	HbA1c ≥6.5%	Test of significance (t-test)	Confidence Interval
1	TG (Mean±SD) mg/dL	138.6±65.3	157.3±71.4	t= -3.5 P=0.001	(-29.3) - (-8.2)
2	CHOL (Mean±SD) mg/dL	176.6±40.5	180.8±46.6	t= -1.2 P=0.2	(-11.0) – (2.5)
3	HDL (Mean±SD) mg/dL	43.7±11.5	42.6±12.3	t= 1.3 P=0.2	(-0.6) – (2.9)
4	VLDL (Mean±SD) mg/dL	27.7±13.1	31.5±14.3	t= -3.5 P=0.001	(-5.8) – (-1.6)
5	LDL (Mean±SD) mg/dL	105.1±31.8	106.8±37.0	t= -0.6 P=0.5	(-7.0) – (3.7)

**Figure 3:**
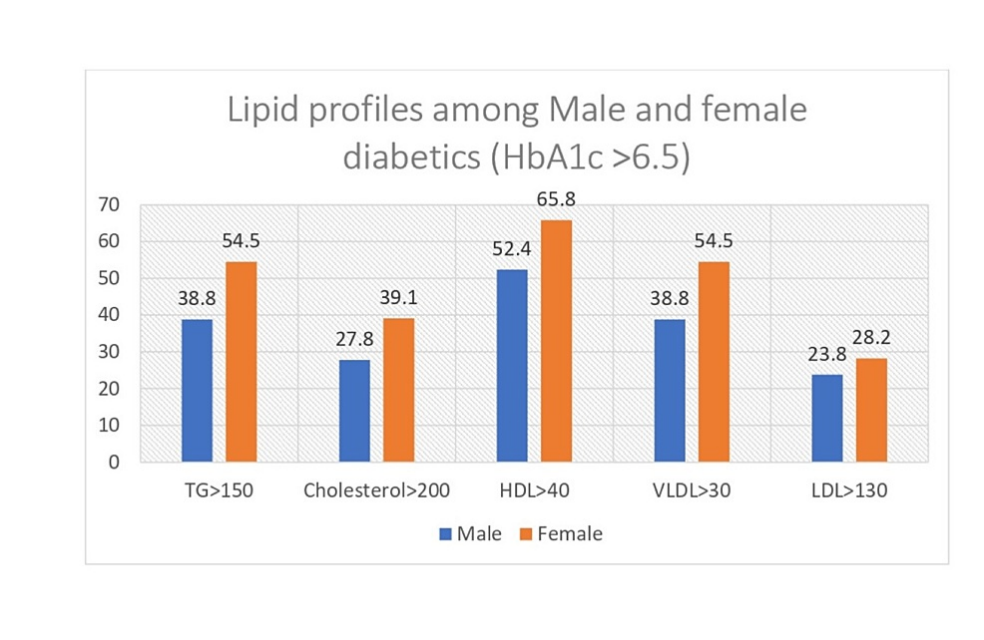
Bar graph showing the lipid profile with borderline high lipid profiles among male and female diabetics (HbA1c > 6.5) TG, triglyceride; HDL, high density lipoprotein; VLDL, very low density lipoprotein; LDL, low density lipoprotein

A higher number of diabetic females had dyslipidemia as compared to diabetic males. The association of gender among diabetics with regard to TG, total cholesterol, VLDL, and HDL was found to be statistically significant (Table 5) Bar graph showing the lipid profile with borderline high lipid profiles among Male and female diabetics (HbA1c >6.5) (Figure 3).

**Table 5:** Lipid profile with desirable vs borderline high lipid profiles among male and female diabetics (HbA1c > 6.5%) TG, triglyceride; HDL, high density lipoprotein; VLDL, very low density lipoprotein; LDL, low density lipoprotein; CHOL, total cholesterol Dyslipidemia-elevation of any parameter of lipid profile (except HDL cholesterol) more than borderline; for HDL if the value is less than 40 mg/dL is considered dyslipidemia.

S. No.	Variable	Male (n=374)	Female (n=202)	Test of Significance (Chi square)
1	TG (mg/dl) Up to 150	229 (61.2)	92 (45.5)	P<0.001
	>150	145 (38.8)	110 (54.5)	
2	CHOL (mg/dl) Up to 200	270 (72.2)	123 (60.9)	P=0.005
	>200	104 (27.8)	79 (39.1)	
3	HDL (mg/dl) >40	196 (52.4)	133 (65.8)	P=0.002
	Up to 40	178 (47.6)	69 (34.2)	
4	VLDL (mg/dl) Up to 30	229 (61.2)	92 (45.5)	P<0.001
	>30	145 (38.8)	110 (54.5)	
5	LDL (mg/dl) Up to 130	285 (76.2)	145 (71.8)	P=0.2
	>130	89 (23.8)	57 (28.2)	

The mean level of TG, total cholesterol, VLDL, LDL, and HDL was higher among diabetic females in comparison to diabetic males. The association between the gender of study subjects and mean levels of TG, total cholesterol, VLDL, and HDL was revealed to be statistically significant in the current study (Table 6). However, no significant association was found between gender and the mean LDL levels of the study participants.

**Table 6:** Mean values of lipid profile as observed in diabetics (HbA1c >6.5%) TG, triglyceride; HDL, high density lipoprotein; VLDL, very low density lipoprotein; LDL, low density lipoprotein; CHOL, total cholesterol

S. No.	Variable	Male (374)	Female (202)	Test of Significance (t test)	95% Confidence Interval
1	TG (mg/dl)	152.3±74.4	166.6±64.4	t= -2.3 P= 0.02	(-26.4) – (-2.0)
2	CHOL (mg/dl)	177.0±46.3	187.9±46.2	t= -2.7 P= 0.007	(-18.8) – (-2.9)
3	HDL (mg/dl)	41.7±12.2	44.2±12.2	t=-2.3 P= 0.02	(-4.5) – (-0.4)
4	VLDL (mg/dl)	30.5±14.9	33.3±12.9	t= -2.3 P= 0.02	(-5.3) – (-0.4)
5	LDL (mg/dl)	104.8±36.4	110.4±37.8	t= -1.7 P= 0.08	(-11.8) – (0.8)

The level of triglycerides was significantly higher among those who had poor glycemic control as compared to those with prediabetics and non-diabetics (ANOVA; F=12.3, p<0.001). Bonferroni’s post hoc analysis revealed a statistically significant difference in mean triglyceride levels between non-diabetics compared to diabetics. A statistically significant difference in triglyceride levels was also found between diabetics and prediabetics. Similarly, the level of VLDL was found to be highest among diabetics followed by prediabetics and non-diabetics. ANOVA analysis revealed this association between diabetic status and VLDL to be statistically significant. On Bonferroni post hoc analysis it was found that there was a statistically significant difference in VLDL levels between diabetic and non-diabetic subjects. Similarly, a statistically significant association was shown by Bonferroni post hoc analysis in VLDL level between diabetics and prediabetics. No statistically significant association was found between diabetic status and any other dyslipidemia parameter (Table 7).

**Table 7:** Association between HbA1c levels with lipid profile (n=1,000) TG, triglyceride; HDL, high density lipoprotein; VLDL, very low density lipoprotein; LDL, low density lipoprotein; CHOL, total cholesterol

S. No.	Variable	Normal (HbA1c<5.7%) N=186	Prediabetics (HbA1c 5.7-6.4%) N=238	Diabetics (HbA1c>6.5%) N=576	ANOVA	Bonferroni post hoc analysis
1	Age (Years)	47.2±14.4	53.9±12.5	52.3±11.9		
2	HbA1c (%)	5.3±0.3	6.1±0.2	9.2±2.6		
3	TG (mg/dl)	133.0±58.5	138.6±65.3	157.3±71.3	F=12.3 P<0.001	A vs C p<0.001 B vs C p= 0.001
4	Cholesterol (mg/dl)	179.0±43.3	176.6±40.5	180.9±46.6	F=0.8 P=0.4	NA
5	HDL (mg/dl)	44.3±12.9	43.8±11.5	42.6±12.3	F=1.7 P=0.2	NA
6	VLDL (mg/dl)	26.6±11.7	27.7±13.1	31.5±14.3	F=12.3 P<0.001	A vs C p<0.001 B vs C p= 0.001
7	LDL (mg/dl)	108.2±34.5	105.1±31.8	106.8±37.0	F=0.4 P=0.7	NA
8	TC/HDL Ratio	4.2±1.1	4.2±1.1	4.2±1.0	F=2.2 P=0.1	NA
9	LDL/HDL Ratio	2.6±0.9	2.5±0.8	2.5±0.8	F=1.4 P=0.2	NA

## Discussion

According to the results of our study, diabetic individuals have a significant prevalence of dyslipidemia. Hypertriglyceridemia (55.7%) and high total cholesterol (31.8%) were the most and least prevalent dyslipidemic parameters seen in our study, respectively. When insulin activity is very low, lipoprotein lipase production is severely inhibited, which significantly impairs the digestion of triglyceride-rich lipoproteins. This causes an increase in triglyceride-rich lipoproteins and a delay in the clearance of chylomicrons and VLDL. A substantial increase in lipolysis in adipose tissue is another effect of insulinopenia that causes the release of free fatty acids into the bloodstream. Higher fatty acid supply to the liver improves triglyceride synthesis in the liver, and increased production and secretion of VLDL are all outcomes of this rise in blood fatty acids. Deterioration of glycemic control in T2DM patients will worsen their underlying dyslipidemia, leading to more significant rises in blood triglyceride levels. LDL levels may rise if fresh VLDL is synthesized at a rate that is sufficiently higher. The production of tiny HDL, which are more vulnerable to rapid clearance, may cause HDL levels to drop. Improvements in glycemic control may also lower LDL levels in persons with diabetes which is very poorly controlled.

In research conducted by Artha et al. [10] involving 140 patients, lipid profile findings like TC, and LDL-C were found to be significantly elevated in patients with poor glycemic control, and this was in concurrence with the findings of our study, though our findings in relation to HDL-C, do not match with that of the given study, as in our study only inverse correlation was noted, which was not statistically significant.

In our study, dyslipidemia was seen to be significantly associated with diabetic females in comparison to diabetic males. Our findings are found to be in concurrence with many other studies where some cardiovascular risk factors, such as HDL-C, total cholesterol, TG, and LDL-C, have shown more dramatic negative alterations brought on by diabetes in females compared to males [11-13]. Recent data from the Strong Heart Study of Native Americans confirm earlier reports that women experience a larger increase in cardiovascular disease due to diabetes than males do. In line with higher triglyceride, very low-density lipoprotein C (VLDL-C), and lower high-density lipoprotein C (HDL-C) levels compared to men in earlier investigations, a higher apoprotein B level was seen in diabetic women in that study. Additionally, higher levels of total and low-density lipoprotein (LDL) cholesterol may be observed [14]. In their investigation of 1,059 type 2 diabetic participants between the ages of 45 and 64, Juutilainen et al. discovered a significantly greater diabetics' relative likelihood of experiencing a catastrophic CHD event in women compared to males, having atherogenic dyslipidemia (low HDL cholesterol and high TG) [15]. But our findings do not coincide with that of a study by Sarfraz, Sajid, and Ashraf, where dyslipidemia was found to be more common among diabetic males [16]. The only limitation of our study was that it was a hospital-based study, so the findings cannot be implemented directly on the general population without conducting a population-based study.

## Conclusions

According to our study, hypertriglyceridemia, and raised very low-density lipoprotein are the most frequent abnormal lipid levels during hyperglycemia-induced dyslipidemia. According to the findings, patients with poor glycemic control are more likely to have dyslipidemia, which may be a major factor in the development of cardiovascular disease in diabetic patients. In contrast to isolated or single abnormalities in the lipid profile, combined lipid abnormalities are more common. To treat this condition, optimal therapy should include regular monitoring of blood glucose levels and lipid profiles.
